# An On-Line Solid Phase Extraction-Liquid Chromatography-Tandem Mass Spectrometry Method for the Determination of Perfluoroalkyl Acids in Drinking and Surface Waters

**DOI:** 10.1155/2015/942016

**Published:** 2015-03-05

**Authors:** Michela Mazzoni, Marianna Rusconi, Sara Valsecchi, Claudia P. B. Martins, Stefano Polesello

**Affiliations:** ^1^IRSA-CNR, Water Research Institute, Via Mulino 19, 20861 Brugherio, Italy; ^2^Thermo Fisher Scientific, 16 avenue du Québec, Silic 765, Villebon-sur-Yvette, 91963 Courtaboeuf Cedex, France

## Abstract

An UHPLC-MS/MS multiresidue method based on an on-line solid phase extraction (SPE) procedure was developed for the simultaneous determination of 9 perfluorinated carboxylates (from 4 to 12 carbon atoms) and 3 perfluorinated sulphonates (from 4 to 8 carbon atoms). This work proposes using an on-line solid phase extraction before chromatographic separation and analysis to replace traditional methods of off-line SPE before direct injection to LC-MS/MS. Manual sample preparation was reduced to sample centrifugation and acidification, thus eliminating several procedural errors and significantly reducing time-consuming and costs. Ionization suppression between target perfluorinated analytes and their coeluting SIL-IS were detected for homologues with a number of carbon atoms less than 9, but the quantitation was not affected. Total matrix effect corrected by SIL-IS, inclusive of extraction efficacy, and of ionization efficiency, ranged between −34 and +39%. The percentage of recoveries, between 76 and 134%, calculated in different matrices (tap water and rivers impacted by different pollutions) was generally satisfactory. LODs and LOQs of this on-line SPE method, which also incorporate recovery losses, ranged from 0.2 to 5.0 ng/L and from 1 to 20 ng/L, respectively. Validated on-line SPE-LC/MS/MS method has been applied in a wide survey for the determination of perfluoroalkyl acids in Italian surface and ground waters.

## 1. Introduction

In the last decade, the worldwide distribution in the different environmental compartments of perfluoroalkyl substances (PFAS) became a concern for the scientific community [1–4]. This class of chemicals has been used in a wide range of industrial and consumer products for the past six decades mainly to repel dirt, water, and oil [5, 6]. PFAS include thousands of chemicals, but the environmental studies have been concentrated mainly on two classes of perfluoroalkyl acids (PFAA), that is, perfluorosulphonic acids (PFSA), which include perfluorooctanesulphonic acid (PFOS), and perfluorocarboxylic acids (PFCA), which include perfluorooctanoic acid (PFOA). PFSA and PFCA are low molecular weight surfactants in which all carbons are bonded to fluorine atoms, consisting of homologous series of molecules that differ in carbon chain length. PFOS and PFOA have been demonstrated to be persistent in the environment and bioaccumulative in the trophic chain. The accumulation in the aquatic trophic chain poses concern about the risks for the end consumers, including humans. After a risk assessment study, the European Commission very recently introduced PFOS in the list of priority hazardous substances which must be monitored in the EU water bodies, setting an environmental quality standard (EQS) of 0.65 ng/L for freshwater and maximum allowable concentrations (MAC) of 36 *μ*g/L and 7.2 *μ*g/L for inland and other surface waters, respectively [7], while the US environmental protection agency (EPA) proposed Provisional Health Advisories of 400 ng/L and 200 ng/L, respectively, for PFOA and PFOS in drinking waters [8]. In Italy, EQSs for five PFAA in surface waters have been recently proposed and are currently under discussion: the lowest EQS is 100 ng/L for PFOA (Valsecchi, personal communication).

The introduction of regulatory restrictions in the use of PFOS and PFOA [9, 10] induced the major PFAS producers to find other substitutes for these compounds especially among the congeners with different chain lengths.

The homologues, which have usually from 4 to 14 carbon atoms (C) for PFCA and from 6 to 10 C for PFSA, show very different physicochemical behaviours which present a serious challenge for the simultaneous determination of PFAA in water samples.

Solubility strongly decreases by increasing the chain length, for example, from 100 g/L for PFHpA to 0.1 g/L for PFUnDA [11, 12], while acidity decreases as the chain length increases (pK_a_s vary from 0.1 to 3.8 in the range PFBA-PFDoDA) ([13] and references therein). The increase of the number of CF_2_ moieties also leads to a significant increase in lipophilicity expressed as pK_ow_; for example, from PFHxA to PFDoDA pK_ow_s increase from 3.68 to 9.21 ([13] and references therein). Generally, for a certain chain length, sulphonates are more lipophilic than carboxylates. All these physicochemical variables drastically influence recoveries with classical extraction techniques such as liquid-liquid extraction (LLE) or solid phase extraction (SPE). According to the review of Jahnke and Berger [14], LLE worked best for PFAA with carbon chain lengths >7 whereas SPE was best suited for PFAA with <10 carbon atoms. Major drawbacks of the SPE approach in PFAA analysis are the sample contamination and possible losses of the surface-active PFAA to container walls and other materials (tubing, connections) besides the problems inherent in SPE such as breakthrough and clogging of the column [14]. Moreover, SPE requires the extraction of large volumes of sample and solvent recovery of the adsorbed molecules followed by solvent concentration and the whole procedure can take some hours.

The automation of SPE has been employed to increase sample throughput for many years [15, 16] and on-line SPE coupled to LC-MS has been also applied to the analysis of PFAA in water samples [17–21]. The on-line SPE made the development of faster methods by reducing the analysis time and thus increasing the analytical productivity possible [16]. The challenge of the on-line SPE methods is still to optimize preconcentration and elution procedures to achieve a satisfactory accuracy in a single run for a class of compounds with different physicochemical variables such as PFAA. In addition, the coupling of on-line SPE with UHPLC is not easy due to the high back pressure generated from the use of high flow rates with a low particle size column (<2 *μ*m). Furthermore, it is common to have release of PFAA (mainly PFOA) from the UHPLC hardware since polytetrafluoroethylene is often used in the manufacturing of tubing and fittings and seals in UHPLC systems. An extra column can be placed before the injector to delay the elution of PFAA from the pump and distinguish between PFAA actually present in the sample and those released by the system. This implies that the system may contain up to 3 columns connected in-line (trap, preconcentration, and chromatographic column).

The aim of our work is to explore the possibility of optimizing an on-line SPE/UHPLC-MS/MS method for PFCA and PFSA ranging, respectively, from 4 to 12 and from 4 to 8 carbon atoms and to understand the factors that influence the method accuracy. The optimized and validated method was then applied to a large survey of Italian surface and drinking waters whose preliminary results are presented in this paper.

## 2. Materials and Methods

### 2.1. Chemicals and Reagents

Perfluorobutanoic acid (PFBA), perfluoropentanoic acid (PFPeA), perfluorohexanoic acid (PFHxA), perfluoroheptanoic acid (PFHpA), perfluorooctanoic acid (PFOA), perfluorononanoic acid (PFNA), perfluorodecanoic acid (PFDA), perfluoroundecanoic acid (PFUnDA), perfluorododecanoic acid (PFDoDA), tetrabutylammonium perfluorobutane sulphonate (PFBS), potassium perfluorohexane sulphonate (PFHxS), and tetrabutylammonium perfluorooctane sulphonate (PFOS) were purchased from Sigma-Aldrich (St. Louis, MO, USA). Separate stock solutions of the analytes were prepared in methanol at a concentration of 1.0 mg/mL of the anionic compound. A multicomponent standard solution containing the 12 analytes at 10 *μ*g/mL was prepared by diluting the stock solutions. Aqueous standard solutions (2–200 ng/L) containing all analytes were freshly prepared by serial dilution of the methanolic mixed solution of standards in clean drinking water. Stable isotope labelled PFCA and PFSA internal standard compounds (SIL-IS) were purchased from Wellington Laboratories (Guelph, ON, Canada) in 2 *μ*g/mL solution mixtures. SIL-IS were ^13^C_4_-PFBA, ^13^C_2_-PFHxA, ^13^C_4_-PFOA, ^13^C_5_-PFNA, ^13^C_2_-PFDA, ^13^C_2_-PFUnDA, ^13^C_2_-PFDoDA, ^18^O_2_-PFHxS, and ^13^C_4_-PFOS. The solution of SIL-IS was diluted to a concentration of 40 ng/mL with methanol. All standard solutions were stored at +4°C. All reagents were analytical reagent grade. Supelco-Supelclean EnviCarb, LC-MS Chromasolv methanol, LC-MS Chromasolv acetonitrile, ammonium acetate (99%), and concentrated formic acid were purchased from Sigma-Aldrich. Water (<18 MΩ cm resistivity) was produced by a Millipore Direct-Q_UV_ water purification system (Millipore, Bedford, MA, USA).

### 2.2. Sample Collection and Pretreatment

**Figure 1:**
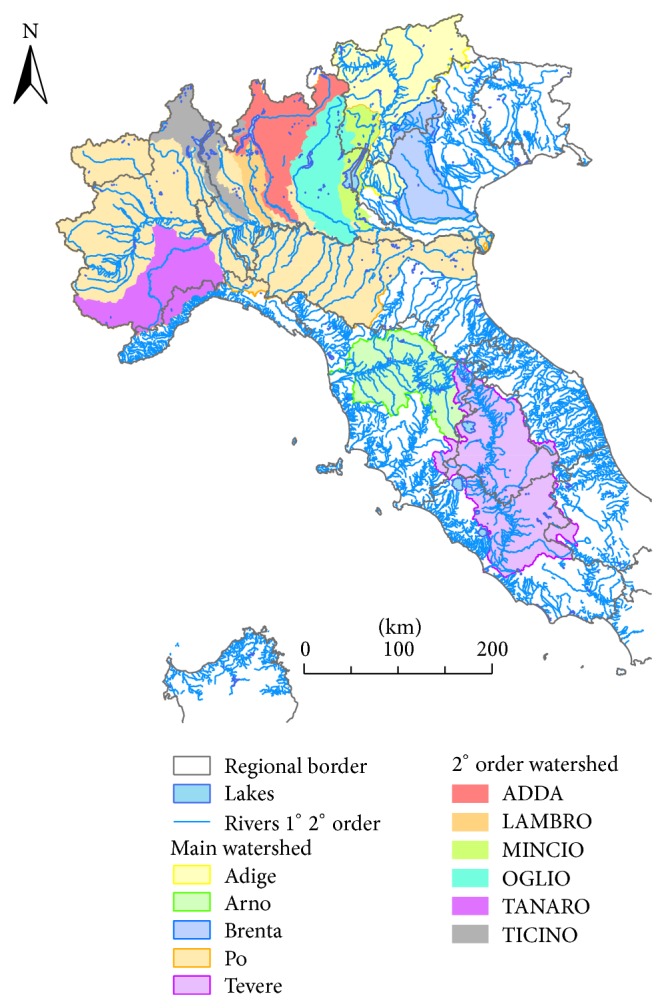
Watersheds of the first and second order rivers which were sampled.

Samples were collected from Italian water bodies between 2010 and 2013. The surveyed river basins were rivers Po, Adige, and Brenta in Northern Italy and rivers Arno and Tevere in Central Italy. In the river Po basin, samples were collected also from the main tributaries Bormida, Tanaro, Ticino, Lambro, Adda, Oglio, and Mincio (Figure 1). River waters were collected by means of a bucket at the centre of the river bed. Samples from lake (lake Como) and transitional environments (lagoons of Venice and Goro in the Po Delta) were directly collected in the sampling vials by manually dipping the vial below the water surface from a boat. Ground waters were also collected between 2010 and 2012 from wells or piezometers.

The samples were collected in polypropylene (PP) centrifuge tubes and kept in a refrigerator at 4°C until analysis. Analyses were carried out within the next 5 days. Contamination or adsorption of target compounds onto tube surfaces was assessed by analysing selected samples (3 drinking water samples with low levels of contamination, 3 river samples with low levels of contamination, and 3 river samples with high levels of contamination) just after collection and after 1 month, without significant differences in the resulting concentrations. In order to prevent the clogging of any parts of the injector or column all aqueous samples were centrifuged. Before injection, in the final analytical procedure, standards and samples were acidified to pH 3 by adding 50 *μ*L of concentrated formic acid to 10 mL of sample directly into the glass vials of the autosampler.

For native spiking experiments, known volumes of the standard mixed solution containing the 12 analytes were added to aqueous sample to give a target concentration of 100 ng/L. For SIL-IS spiking experiments, 25 *μ*L of the diluted SIL-IS solution was added to native-spiked aqueous samples to obtain concentrations of 100 ng/L both of native and of stable isotope labelled perfluorinated compounds.

### 2.3. On-Line SPE and Analytical Separation

**Figure 2:**
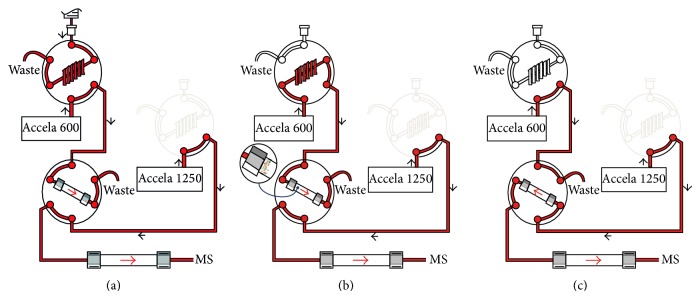
Schematic representation of the on-line SPE system used. (a) Loading of the sample into the high volume loop. (b) Transfer of the sample from the injection loop to the preconcentration column. (c) Transfer of the analytes retained in the preconcentration column to the chromatographic column.

The on-line SPE has been carried out by a Thermo EQuan system which consists of two Thermo Scientific Accela LC pumps (600 and 1200) with a preconcentration column (Thermo Hypersil GOLD aQ 12 *μ*m, 20 × 2.1 mm), an analytical column (Thermo Hypersil GOLD PFP 1.9 *μ*m, 50 × 2.1 mm), and a CTC PAL autosampler equipped with three 6-way VICI valves (Figure 2). Samples were injected into a high volume loop (Figure 2(a)) and then transferred onto the preconcentration column by the loading pump (Thermo Scientific Accela 600) using 2 mM ammonium acetate (NH_4_OAc) with 5% methanol (MeOH) eluent at 1200 *μ*L/min (Figure 2(b)). When the enrichment step was completed (260 s), a 6-way valve on the autosampler switched over and the elution pump (Accela 1200) flowed the elution gradient, composed of two eluents [(A) 2 mM NH_4_OAc-5% MeOH and (B) methanol] at 300 *μ*L/min, through the preconcentration column and the analytical column (Figure 2(c)). The loading and the elution gradients are illustrated in Table 1. In order to delay the interfering background peaks of perfluorinated compounds, which are present in solvents or are released from the analytical system, a trap column (Thermo Hypersil GOLD 1.9 *μ*m, 50 × 2.1 mm) has been placed between the analytical pump and the injection valve.

### 2.4. Mass Spectrometry

A triple quadrupole mass spectrometer (Thermo Scientific TSQ Quantum Access MAX) equipped with a heated-electrospray ionization (HESI-II) probe was used. The source-dependent parameters were as follows: spray voltage (3000 V); sheath gas pressure (25 psi); auxiliary gas pressure (10 arbitrary units); skimmer offset (0 V), ion transfer tube temperature (270°C), vaporizer temperature (40°C); high purity argon (>99.98%) which was used as the collision gas (1.5 mTorr). The mass spectrometer operated at a resolution of 0.7 Da in negative selected reaction monitoring (SRM) mode.  Table 2 lists the MS/MS transitions, tube lens offset, and collision energies applied for the different target analytes and isotope labelled standards. The Xcalibur 2.1 (Thermo Scientific) was used for instrument control, data acquisition, and processing.

### 2.5. Confirmation and Quantification

Analytes were identified by comparing their retention times (RT) with the RT of the SIL internal standards (deviation ≤ ±0.25%) when possible and with the RT of the reference standards if no SIL internal standard was available. For all the analytes, except PFBA, one precursor and two product ions were monitored; PFBA does not fragment into two stable product ions and only one precursor and one product ion were monitored (Table 2).

Separate stock solutions of the analytes were prepared in methanol at a concentration of 1.0 mg/mL. A standard mixed solution in methanol containing the 12 selected analytes at 5 or 10 *μ*g/mL was made from the stock solutions. The SIL-IS solution was diluted to 40 ng/mL with methanol. All standard solutions were stored at 4°C. Calibration curves were prepared using aqueous standard solutions containing all analytes. The aqueous standard solutions were freshly prepared by serial dilution of the methanol standard mixed solution in real drinking water which was devoid of analysed contaminants. Quantification was performed by using the isotopic dilution method and calibration curves were made before each analytical run. Before injection, standards and samples were acidified to pH 3 and spiked with SIL-IS by adding 50 *μ*L of concentrated formic acid and 25 *μ*L of the diluted SIL-IS solution (40 ng/mL) to 10 mL of sample.

Blank samples (clean drinking water) were injected at the beginning and at the end of the analytical sequence and every ten samples. No carryover and contamination were detected.

Applicability to different typologies of waters, including saltwater and wastewater, was tested through fortifications of the different sample matrices. Relative recoveries were in the same range calculated during validation procedures (75 and 135%).

The performance of the on-line-SPE-UHPLC-MS/MS method was verified in the “PFOS and PFOA in surface water” proficiency test organised in 2013 by the PT-WFD network (http://www.pt-wfd.eu). Analytical results were satisfactory with calculated *z*-scores of +1.43 and +1.70 and expanded relative uncertainty of 13% and 10% for PFOS and PFOA, respectively.

## 3. Results and Discussion

### 3.1. Method Development

In an on-line SPE procedure, several experimental variables, such as sample volume and sample pH, should be optimized in order to achieve the maximum extraction efficiency of the target analytes. Generally, it may be difficult to achieve comparable and reliable efficiencies for all target compounds when the analytes have a broad range of polarity. In order to get the maximum chromatographic efficiency the trapped analytes should be eluted and refocused onto the analytical column by the analytical elution gradient by the time the extraction column is switched into the analytical flow path. However in multiresidue analysis, the gradient elution for reversed-phase separations usually starts at high percentage of aqueous in the mobile phase, and the slow elution from the SPE preconcentration column results in peak broadening, which may cause a decrease in efficiency and thereby in sensitivity [22, 23]. For these reasons, before carrying out the method validation, the optimisation of the elution gradient and the volume as well as the matrix modification of the injected sample was carried out in order to achieve the best recovery and sensitivity for the on-line SPE/UHPLC-MS/MS analysis of the perfluorinated carboxylic and sulphonic acids.

#### 3.1.1. Optimisation of the Elution Gradient

Optimisation of the analytical separation was carried out by direct injection of standard solutions with a 25 *μ*L loop. The elution gradient started at only 5% of mobile phase B (methanol) in order to separate the shorter chain PFCA (PFBA and PFPeA). An initially faster gradient with mobile phase B raising from 5 to 70% within 2 min followed by a slower gradient with mobile phase B raising up to 100% within 5 min allowed the separations of all perfluoroalkyl acids and the elution of the longer chain homologues (PFUnDA and PFDoDA) within 5 min. Then, an isocratic step at 100% methanol for 3.5 min was followed by a fast return to 95% of mobile phase A in 1 min and a conditioning step of 4 min at starting composition before the following analysis.

In the on-line SPE method, during the sample loading step, analytes are trapped in the stationary phase of the preconcentration column. Then, elution of analytes is achieved in back-flush mode by putting in-line the preconcentration column with the eluting mobile phase.

The first approach was simply to use in the on-line procedure the separation gradient elution program, as optimised by direct injection of analytes. In this case (Table 1: “unchanged gradient”) the gradient program started soon after the end of the loading of the sample into the preconcentration column (4.34 min). Throughout the so-called loading time, a solvent mixture at the initial conditions (5% methanol) isocratically flowed through the chromatographic column. By using these settings, peak broadening and distortion were observed for the shorter chain and more polar homologues (PFBA and PFPeA), because these compounds, which show lower affinity for the preconcentration column stationary phase, were poorly focused on the preconcentration column.

To overcome this problem, the “solvent plug injection technique” [22] was implemented. This technique provides elution bands of only a few seconds width using high percentage solvent in order to get a rapid transfer of the analytes from the preconcentration column to the analytical column as well as to keep them focused. Gode et al. [22] generated short plugs of high-elution strength solvent by means of an external loop and an additional LC-pump. We achieved the same high-elution strength solvent plugs by using the separation UHPLC pump which is characterised by very low dead volumes. A step with a high percentage of organic solvent (80% methanol) was inserted in the eluting gradient at the switching time, in order to provide narrow high-elution strength eluent band containing all the eluted analytes (Table 1: “plug gradient”). The subsequent elution gradient was modified in order to obtain a chromatographic separation similar to the one used for direct injection of the sample (Table 1).

**Figure 3:**
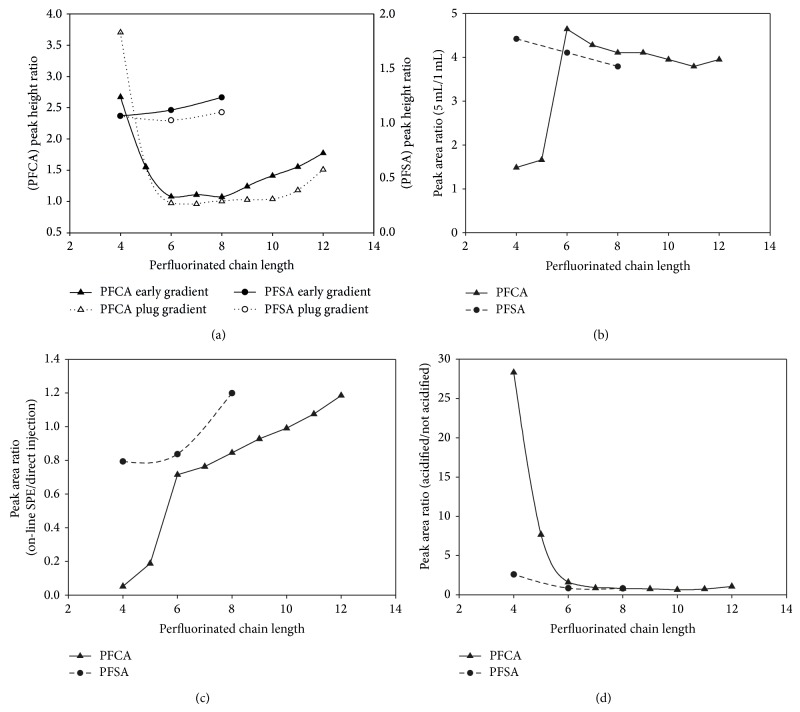
Development of the on-line SPE method. (a) Effect of different elution gradients, “plug gradient” or “early gradient” compared with “unchanged gradient,” on the analyte peak height; injection of 5 mL of acidified aqueous standard at 200 ng/L. (b) Effect of the sample volume on the peak area; injection in “early gradient” mode of aqueous standard at 200 ng/L. (c) Extraction efficiency of the 5 mL on-line SPE injection volume; on-line SPE injection in “early gradient” mode of 5 mL of aqueous standard at 200 ng/L and direct injection of 25 *μ*L of aqueous standard at 40 *μ*g/L. (d) Effect of acidification of the sample on the peak area; injection in “early gradient” mode of aqueous standard at 200 ng/L.

The effects of the different elution gradient are reported in Figure 3(a) which shows the height ratios between the analyte peaks detected with the plug gradient and those obtained with the unchanged gradient. Both early- and late-eluting perfluorocarboxylic acids benefited from “plug gradient” by improving the shape of their peaks whereas no significant effects were detected for PFSA. The peak heights improved up to 3.7 times for the less retained PFCA. Nevertheless, the optimization of the “solvent plug injection technique” is laborious and highly time-consuming because it involves several runs to find out the best high-elution strength solvent and elution band width as well as the subsequent elution gradient in order to avoid loss of retention along with severe peak distortion and artefact formation [22].

Finally, we tested a further and simpler approach: when the loading on preconcentration column is still on going, we anticipated the start of the gradient program in separation column, and, in this way, when the mobile phase is switched on the preconcentration column, the mobile phase had an higher solvent percentage and thereby is able to quickly transfer analytes onto the separation column with a focusing effect (Table 1: “early gradient”). The optimum mobile phase composition at the switching time is determined by calculating the composition when the first peak (PFBA) elutes in the chromatographic run obtained by direct injection of the standard mixture without a preconcentration step. Improvements in the PFAA peak heights with respect to the “unchanged gradient” were analogous to those obtained by the “injection plug” technique and even better for the longer chain PFCA (Figure 3(a)). Because of its simplicity, the “early gradient” was chosen for the rest of the optimization procedure.

#### 3.1.2. Sample Volume

Sample volume effect was evaluated comparing the peak areas obtained by injecting 1 and 5 mL of the highest standard mixture (200 ng/L). Proportionality was satisfactory (Figure 3(b)), with an average response ratio of 4.1 for PFSA and PFCA from 6 to 12 carbon atoms, despite a slight peak broadening. Only PFBA and PFPeA did not show any proportional improvement in the response when the loop volume increased, suggesting that the shorter homologues in the perfluorocarboxylic acid series have smaller breakthrough volumes on the concentration column.

The loss in efficiency attributable to the on-line preconcentration step was evaluated by comparing the peak areas obtained in the on-line SPE (injection volume: 5 mL) with those obtained by direct injection (injection volume: 25 *μ*L) of the same analyte mass (100 pg). As shown in Figure 3(c) ratios of peak areas close to 1 (0.75–1.2) were obtained for all PFSA homologues and the PFCA with carbon chain length greater than 6, indicating an efficient extraction for those compounds. Differently, the response of the more soluble PFCA (PFBA and PFPeA) in the on-line SPE method was much lower than that obtained by direct injection (0.1–0.2) because of their limited affinity for the stationary phase of the preconcentration column.

#### 3.1.3. Chemical Modification of the Sample before Injection

Since all target analytes are acid compounds, samples were acidified to pH 3 by adding 50 *μ*L of concentrated formic acid in order to improve their retention on the endcapped C18 phase of the preconcentration column. Acidification significantly improved the peak areas of the less retained and more soluble homologues (PFBA, PFPeA, PFHxA, and PFBS) whereas no effects were pointed out for the homologues with a longer carbon chain (Figure 3(d)). This can be explained by the fact that the acidification suppresses the ionization of the PFCA, and thereby increases their affinity for the reverse stationary phase of the preconcentration column.

The effect on the LC-MS/MS response for the PFAA was also examined as a function of the percentage of solvent (acetonitrile and methanol) modifier in the water samples. Addition of organic solvent (10%) to the sample before the injection caused a decrease in the area response for shorter and longer chain perfluorocarboxylic homologues (peak area ratio ranging from 0.65 to 0.75). However, this effect was not observed when injecting 25 *μ*L of the aqueous standards directly onto the analytical column without preconcentration step: in the latter case peak areas and symmetry increased with perfluorinated carbon chain when the standard was prepared in water and methanol. The negative effect on response of the solvent addition procedure in on-line SPE may be attributed to a loss of retention on the preconcentration column. Finally, the optimised procedure was to acidify the sample at pH 3 without further matrix modification.

### 3.2. Method Validation

The optimised method (early gradient), with 5 mL injection volume of acidified samples, was subjected to the validation procedure.

#### 3.2.1. Matrix Effect and Mutual Suppression Effect

**Figure 4:**
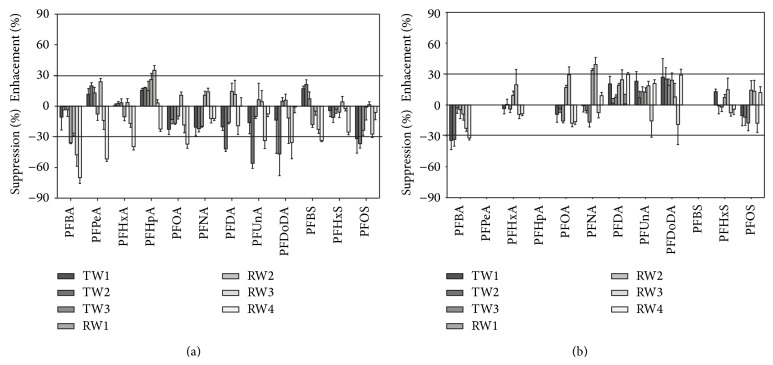
Not corrected (a) and corrected by SIL-IS (b) total matrix effects (%). TW1 and TW2 are drinking water samples produced from ground water; TW3 is a drinking water sample produced from lake water. RW1 and RW3 are water samples collected in low polluted rivers; RW2 and RW4 are water samples collected in a river impacted by industrial discharges and in an organic polluted river, respectively. Spiked concentration of native and coeluting SIL-IS at 100 ng/L level.

LC-ESI-MS/MS is the most suitable analytical technique for the analysis of the target compounds though it may suffer from matrix effects on the ionization efficiency ([24] and references therein, [25]). Matrix effects on the ionization in ESI-MS/MS determination are usually evaluated by comparing the signal responses of target compounds in matrix extracts, spiked immediately before instrumental analysis, with those in standard solutions. But when the extraction step is on-line connected with the HPLC separation the effects of sample matrix on extraction recovery cannot be evaluated separately from the matrix effects on efficiency of ionization. Total matrix effects in the on-line SPE analysis of target compounds were determined analysing native-spiked aqueous samples at a 100 ng/L level. The experiments were carried out with different matrices: drinking water produced from ground water (TW1 and TW2) and from lake water (TW3), surface water from rivers with a low level of pollution (RW1 and RW3), and rivers impacted by industrial discharges (RW2) and by organic domestic wastewaters (RW4).  Figure 4(a) shows the percentage recovery of the spiked native standards. Signal response suppressions were more frequent than the enhancements. Total matrix effects ranged between −70 and +35% with more than 80% of the results included in the range ±30%. Suppression of the signal response of the target compounds was found in some samples, both drinking and river waters, for short, as well as long carbon chain perfluorinated homologues (Figure 4). We tested the possibility to remove the matrix by dispersive SPE clean-up with activated graphitized carbon (ENVI-Carb), which has been successfully employed to purify methanol and acetonitrile extracts for PFAA analysis [26], but very low recoveries of the target analytes in aqueous samples (results not shown) were achieved. We explored, thus, the utilisation of isotope dilution with SIL-IS analogues to correct matrix effects on ionization efficiency as well as to compensate for variation in injection, sample extraction, and instrumental parameters.

**Figure 5:**
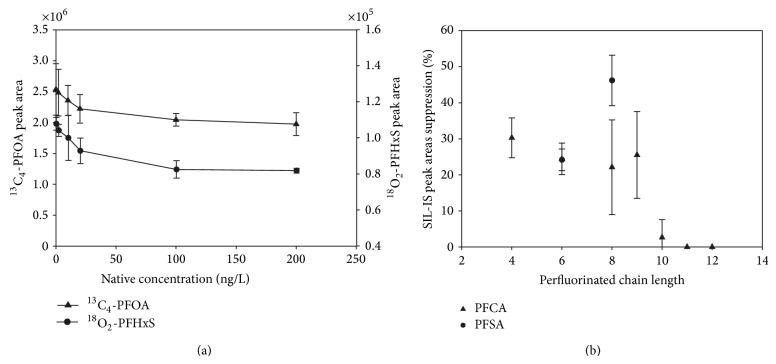
Effect of native concentration on coeluting SIL-IS peak area. (a) Injection in “early gradient” mode of 5 mL of acidified aqueous standards spiked with 100 ng/L of SIL-IS. (b) Extent of suppression of SIL-IS peak area at 100 ng/L was calculated as SIL-IS peak area of 0 ng/L native standard solution minus SIL-IS peak area of 200 ng/L native standard solution/SIL-IS peak area of 0 ng/L native standard solution X 100.

For some target compounds, the peak area of the coeluting SIL-IS decreased with increasing native analyte concentrations.  Figure 5(a) shows the PFOA and PFHxS results obtained in the analysis of calibration standards spiked with SIL-IS at 100 ng/L level. This effect was also observed with a 25 *μ*L direct injection, without the preconcentration step, indicating that the effect is not due to a competition on the active sites in the preconcentration column but it is really a suppression effect in the electrospray ionization. Previous reports have noted mutual ionization suppression between target analytes and their coeluting SIL-IS [27, 28] which was attributed to the competition among ions for the limited number of excess charge sites on the generated droplet during electrospray ionization [29, 30]. Liang et al. [28] investigated this phenomenon for nine drugs: generally, the extent of suppression in each drug-SIL-IS pair was concentration-dependent and was correlated with the hydrophobicity of the compounds.

Maximum suppression was determined for each perfluorinated SIL-IS at 200 ng/L native concentration (Figure 5(b)). The suppression of PFCA decreases with the increase of the number of carbon chain length, but the suppression of PFSA is lower for the shorter chain homologues. Quantitation, however, was not affected by the variation in the peak area of the internal standard. Mutual suppression effects of target analytes and SIL-IS are equal and this ensures good linearity of peak area ratio versus analyte concentrations [27]. Results of Figure 4(a) were recalculated using SIL-IS calibrations (Figure 4(b)). The scattering of the results was significantly reduced: total matrix effects ranged between −34 and +39%. SIL-IS successfully corrected the effects on the ionization as well as any eventual losses during the on-line extraction step.

#### 3.2.2. Linearity and Sensitivity

The linearity of the instrumental response was assessed by injecting five levels of aqueous standards with native analyte concentrations spanning two orders of magnitude (1, 5, 10, 50, and 100 ng/L for PFUnDA and PFDoDA and 2, 10, 20, 100, and 200 ng/L for the remaining compounds) spiked with SIL-IS at 100 ng/L level. Working calibration curves presented acceptable linearity range and coefficients of determination (*R* ^2^) higher than 0.95 for all target compounds (Table 3). Deviations from the linearity occurred at the highest concentrations where mutual suppression with SIL-IS is likely to occur (see Section 3.2.1). Nevertheless, most of the measured concentrations fell into the linear part of the calibration curves.

Limits of detection (LOD) and limits of quantification (LOQ) were estimated as threefold and tenfold, respectively, the standard deviation of the aqueous standard solutions, prepared in real drinking waters, at the lowest injected concentrations (1 and 2 ng/L), according to the ISO 6107-2: 2006 standard. LODs and LOQs ranged from 0.2 to 5.0 ng/L and from 1 to 20 ng/L, respectively (Table 3). It is important to emphasize that these values refer to the whole analytical procedure, incorporating mutual ionization suppression and recovery losses, while the detection limits estimated from solvent calibration curves usually underestimate the true values.

#### 3.2.3. Accuracy

Method precision has been evaluated by 3 replicate injections of standards prepared in real drinking waters in the 1–100 ng/L range for PFUnDA and PFDoDA and 2–200 ng/L range for the other compounds on 4 nonconsecutive days (Table 3). Repeatabilities (intraday precision) and reproducibilities (interday precision) were under 20% for carboxylic acid homologues from 7 to 9 carbon atoms while PFBS, PFHxS, PFOS, PFBA, PFPeA, PFHxA, PFDA, PFUnDA, and PFDoDA showed RSDs from 30 to 65% at the lower limits of the explored range which was very close to their detection limits. Trueness is expressed as recoveries determined by analysing spiked drinking and river waters since no certified reference materials were available for PFAA in these matrices. The recoveries were between 76 and 134% for PFCA and between 87 and 115% for PFSA (Table 3).

## 4. Applications to Real Samples

The validated on-line SPE/LC-MS/MS methodology has been applied in a wide survey of the PFAA concentrations in Italian surface and drinking waters with the aim to get a reliable picture of PFAA contamination and sources in Italian waters. The low volumes needed for the on-line extraction allowed us to minimise the duration of the sampling campaigns, avoid the need for a large refrigeration system during the travel, and thus minimise the risk of sample degradation and contamination during the transport to the laboratory.

Data summarized in Table 4 showed that the method can be applied to the monitoring of different typologies of waters, including complex matrices, such as saline waters of coastal lagoons and discharges from industrial and urban wastewater treatment plants (WWTPs).

Results of monitoring of Italian surface waters have been recently discussed by Valsecchi et al. [31].

Monitoring data of rivers in Northern Italy, which includes rivers in the basins of river Po, Adige, and Brenta which discharges into the Adriatic Sea, show that the highest concentrations were measured for PFOA and PFBS, while the highest detection frequencies were found for PFOA, PFHxA, and PFBS. The highest concentrations were measured downstream the discharges of perfluorinated compounds and polymers factories, but the detection frequency suggests that the same compounds were diffused in many rivers of the explored territories.

Rivers in Central Italy (belonging to the basins of Tevere and Arno rivers) were less impacted and the only substances measured at concentrations >100 ng/L were PFOA and PFBS.

PFHxA, PFOA, and PFOS were detected at the highest detection frequency in ground waters used for drinking water abstraction (Table 4) and these findings reflected diffuse and historical pollution coming both from urban pressure and industrial pressure on the territory. In fact, the highest concentrations were measured for PFOS (up to 121 ng/L) which was much diffused since the 80s, but it is currently subject to restrictions in use [9]. Nevertheless, it should be underlined that all measured concentrations were significantly lower than the Provisional Health Advisories of 400 ng/L and 200 ng/L proposed by US EPA, respectively, for PFOA and PFOS in drinking waters [8], showing a very limited risk for the final consumer.

The validated on-line SPE-UHPLC-LC/MS/MS method has been successfully used also for determining PFAA in difficult matrices. We were able to determine low concentrations of PFAA in transitional waters, characterised by a range of salinity from 23 to 31‰, collected in the lagoons of Venice and in the Po Delta. We also analysed effluents from industrial and urban WWTPs and, after the appropriate dilution, concentrations up to 4.8 mg/L of PFBS and 0.7–0.8 mg/L for PFOA and PFHxA were measured. Samples were injected in the system after centrifugation and dilution and matrix effects were minimized by using internal isotopically labelled standards.

## 5. Conclusions

The use of on-line SPE coupled with UHPLC, with 2 *μ*m particle size columns, made the development of faster methodology by reducing the analysis time and thus increasing the sample throughput possible. However, reduction of the total analysis time originating from the development of ultrafast separation and the reduced sample treatment may introduce new analytical challenges during method development, such as increase of ionization matrix effects and loss of chromatographic efficiency.

In this work, we present the development and validation of a rapid analytical method for the simultaneous determination of compounds with different polarities and other physicochemical properties such as PFCA and PFSA. Manual sample preparation was reduced to sample centrifugation and acidification, thus eliminating several procedural errors and contamination and significantly reducing costs and analytical time, which was only about 20 min from extraction to analysis.

Method development was carried out after having understood the factors that can negatively impact the on-line SPE methodology. Optimization of elution gradient and acidification of the aqueous samples allowed reliable chromatographic efficiencies for all compounds, including shorter chain perfluorocarboxylic acids such as PFBA and PFPeA. Mutual ionization suppression between target perfluorinated analytes and their coeluting SIL-IS was reported for the first time for perfluorinated compounds. Generally, the extent of suppression in each target compound-SIL-IS pair was correlated with the carbon chain length. Validated on-line SPE-LC-MS/MS method has been applied in a wide survey of the concentrations of PFAA in Italian surface and drinking waters. The method is able to measure contaminants at the environmental levels in urbanized and industrialized areas and attain most of the adopted quality standards except for European EQS for PFOS (0.65 ng/L) which is considered a challenging limit for every LC-MS analytical method.

## Figures and Tables

**Table 1:** Elution gradients used by the loading pump and the elution pump. Elution pump flowed at 300 *µ*L/min. Loading time was 260 s. Sample volume was 5 mL.

Time (min)	Elution pump (*unchanged gradient*)		Elution pump (*plug gradient*)		Elution pump (*early gradient*)		Loading pump		
	A%	B%	A%	B%	A%	B%	Flow (*µ*L/min)	A%	B%
0	95	5	95	5	95	5	1200	100	0
3.00					95	5			
3.99			95	5					
4.00			20	80					
4.34	95	5							
4.50			20	80			1200	100	0
4.51			65	35					
4.75			55	45					
5.00			30	70	30	70			
6.00			30	70					
6.34	30	70							
6.50							200	10	90
10.00					0	100			
11.00			0	100					
11.34	0	100							
11.50							200	10	90
13.50					0	100			
14.50			0	100	95	5	1200	100	0
14.84	0	100							
15.50			95	5	95	5			
15.84	95	5							
16.50	95	5	95	5			1200	100	0

(A) 2 mM ammonium acetate with 5% methanol.

(B) Methanol.

**Table 2:** LC/MS/MS parameters for all target analytes and internal standards.

Compound	Abbreviation	Precursor ion	Product ion		
		(*m*/*z*)	(*m*/*z*)	Collision energy	Tube lens offset
Target analytes					
Perfluorobutanoic acid	PFBA	212.9	168.9	11	80
perfluoropentanoic acid	PFPeA	262.9	69.0	39	85
			218.9	11	85
perfluorohexanoic acid	PFHxA	312.9	119.1	22	86
			268.9	11	86
perfluoroheptanoic acid	PFHpA	362.9	169.0	18	91
			318.9	12	91
perfluorooctanoic acid	PFOA	412.9	169.0	19	96
			368.9	13	96
perfluorononanoic acid	PFNA	462.9	218.9	18	104
			418.9	13	104
perfluorodecanoic acid	PFDA	512.9	268.9	18	97
			468.9	13	97
perfluoroundecanoic acid	PFUnDA	562.9	268.8	20	96
			518.8	14	96
perfluorododecanoic acid	PFDoDA	612.9	318.8	20	107
			568.9	14	107
Perfluorobutane sulphonate	PFBS	298.9	80.2	44	85
			99.1	32	85
Perfluorohexane sulphonate	PFHxS	398.9	80.1	38	91
			99.0	34	91
Perfluorooctane sulphonate	PFOS	498.9	80.3	45	104
			99.1	45	104
Internal standards					
Perfluoro-*n*-[^13^C_4_] butanoic acid	^ 13^C_4_-PFBA	216.9	171.9	11	80
Perfluoro-*n*-[^13^C_2_] hexanoic acid	^ 13^C_2_-PFHxA	314.9	269.9	11	86
Perfluoro-*n*-[^13^C_4_] octanoic acid	^ 13^C_4_-PFOA	416.9	371.9	13	96
Perfluoro-*n*-[^13^C_5_] nonanoic acid	^ 13^C_5_-PFNA	467.9	422.9	13	104
Perfluoro-*n*-[^13^C_2_] decanoic acid	^ 13^C_2_-PFDA	514.9	469.9	13	97
Perfluoro-*n*-[^13^C_2_] undecanoic acid	^ 13^C_2_-PFUnDA	564.9	519.8	14	96
Perfluoro-*n*-[^13^C_2_] dodecanoic acid	^ 13^C_2_-PFDoDA	614.9	569.9	14	107
Perfluoro-*n*-hexane [^18^O_2_] sulphonate	^ 18^O_2_-PFHxS	402.9	103.0	34	91
Perfluoro-*n*-octane [^13^C_4_] sulphonate	^ 13^C_4_-PFOS	502.9	99.1	45	104

**Table 3:** Validation parameters (injection in “early gradient” mode of 5 mL of acidified standards and samples).

	Linearity^a^		Precision (RSD%)^b^		Trueness (%)^c^		Sensitivity (ng/L)	
	(ng/L)	*R* ^2^	Intraday	Interday	TW	RW	LOD	LOQ
PFBA	2–200	0.955	7–27	10–33	76	83	5.0	20
PFPeA^*^	2–200	0.962	10–32	10–28	115	88	2.0	4
PFHxA	2–200	0.969	2–8	8–33	98	103	0.2	1
PFHpA^*^	2–200	0.970	2–9	6–14	116	111	0.2	5
PFOA	2–200	0.963	1–13	8–12	91	103	0.5	3
PFNA	2–200	0.960	4–12	10–16	92	119	0.5	1
PFDA	2–200	0.985	10–20	16–39	110	119	0.5	1
PFUnDA	1–100	0.972	10–31	24–53	119	109	0.5	1
PFDoDA	1–100	0.971	10–35	30–65	134	112	1.0	2
PFBS^*^	2–200	0.990	6–28	11–44	115	80	1.0	10
PFHxS	2–200	0.970	4–13	10–25	103	103	5.0	20
PFOS	2–200	0.989	8–49	14–48	87	105	2.5	10

^*^Values not corrected by SIL-IS; ^a^linearity was calculated with the analysis of five levels of aqueous standard solutions; ^b^precision is calculated by injecting standards in the 1–100 ng/L range for PFUnDA and PFDoDA and 2–200 ng/L range for the other target compounds (intraday: *n* = 3; interday: *n* = 12); ^c^trueness is expressed as recoveries determined by analysing native-spiked tap waters (TW) and river waters (RW) at 100 ng/L level.

**Table 4:** Summary of PFAS concentrations in Italian surface and ground waters.

Samples	Monitoring period	PFBA	PFPeA	PFHxA	PFHpA	PFOA	PFNA	PFDA	PFUnDA	PFDoDA	PFBS	PFHxS	PFOS
Rivers in Northern Italy	2010–2013												
(number of samples = 222)	Min (ng/L)	<LOD	<LOD	<LOD	<LOD	<LOD	<LOD	<LOD	<LOD	<LOD	<LOD	<LOD	<LOD
	Median (ng/L)	<LOD	<LOD	4	2	28	<LOD	<LOD	<LOD	<LOD	3	<LOD	3
	Max (ng/L)	411	974	892	946	6.480	174	99	58	19	4.328	36	218
	% positive sample	28	48	75	64	88	46	43	25	16	65	4	55

Rivers in Central Italy	2011–2013												
(number of samples = 26)	Min (ng/L)	<LOD	<LOD	<LOD	<LOD	<LOD	<LOD	<LOD	<LOD	<LOD	<LOD	<LOD	<LOD
	Median (ng/L)	16	<LOD	3	2	14	2	<LOD	<LOD	<LOD	<LOD	<LOD	3
	Max (ng/L)	79	15	40	29	222	30	51	11	4	335	<LOD	34
	% positive sample	73	31	58	69	73	69	35	12	8	42	0	62

Transitional waters	2011–2013												
(number of samples = 14)	Min (ng/L)	<LOD	<LOD	1	<LOD	1	<LOD	<LOD	<LOD	<LOD	1	<LOD	<LOD
	Median (ng/L)	<LOD	2	2	<LOD	5	<LOD	1	1	2	5	<LOD	4
	Max (ng/L)	31	8	4	1	19	<LOD	1	1	3	10	<LOD	9
	% positive sample	14	64	100	7	100	0	64	79	93	100	0	57

WWTP effluents	2011–2013												
(number of samples = 18)	Min (ng/L)	<LOD	<LOD	<LOD	1	3	<LOD	<LOD	<LOD	<LOD	<LOD	<LOD	<LOD
	Median (ng/L)	<LOD	<LOD	38	22	78	16	14	4	<LOD	<LOD	<LOD	<LOD
	Max (ng/L)	<LOD	582.881	811.431	171.231	712.877	1.080	1.414	1.128	4	4.833.734	<LOD	18.688
	% positive sample	0	44	72	100	100	94	94	78	39	17	0	28

Ground waters	2010–2012												
(number of samples = 77)	Min (ng/L)	<LOD	<LOD	<LOD	<LOD	<LOD	<LOD	<LOD	<LOD	<LOD	<LOD	<LOD	<LOD
	Median (ng/L)	<LOD	<LOD	0.3	<LOD	<LOD	<LOD	<LOD	<LOD	<LOD	<LOD	<LOD	<LOD
	Max (ng/L)	20	22	37	31	53	13	16	9	13	29	45	121
	% positive sample	67	29	48	40	47	17	16	12	16	38	22	45

## References

[1] Giesy J. P., Kannan K. (2001). Global distribution of perfluorooctane sulfonate in wildlife. *Environmental Science and Technology*.

[2] Giesy J. P., Kannan K. (2002). Perfluorochemical surfactants in the environment. *Environmental Science and Technology*.

[3] Kannan K. (2011). Perfluoroalkyl and polyfluoroalkyl substances: current and future perspectives. *Environ. Chem*.

[4] Lindstrom A. B., Strynar M. J., Libelo E. L. (2011). Polyfluorinated compounds: past, present, and future. *Environmental Science and Technology*.

[5] Prevedouros K., Cousins I. T., Buck R. C., Korzeniowski S. H. (2006). Sources, fate and transport of perfluorocarboxylates. *Environmental Science and Technology*.

[6] Buck R. C., Franklin J., Berger U. (2011). Perfluoroalkyl and polyfluoroalkyl substances in the environment: terminology, classification, and origins. *Integrated Environmental Assessment and Management*.

[7] European Commission (2013). Directive 2013/39/EU of the European Parliament and of the Council of 12 August 2013 amending Directives 2000/60/EC and 2008/105/EC as regards priority substances in the field of water policy. *Official Journal of the European Union*.

[8] US Environmental Protection Agency (2009). *Provisional Health Advisories for Perfluorooctanoic Acid (PFOA) and Perfluorooctane Sulfonate (PFOS)*.

[9] European Commission (2006). Directive 2006/122/EC of the European Parliament and of the Council of 12 December 2006 amending for the 30th time Council directive 76/769/EEC on the approximation of the laws, regulations and administrative provisions of the Member States relating to restrictions on the marketing and use of certain dangerous substances and preparations (perfluorooctane sulfonates). *Official Journal of the European Union*.

[10] US Environmental Protection Agency http://www.epa.gov/opptintr/pfoa/pubs/stewardship/pfoastewardshipbasics.html.

[11] Jensen A. A., Poulsen P. B., Bossi R. (2008). *Survey and Environmental/Health Assessment of Fluorinated Substances in Impregnated Consumer Products and Impregnating Agents*.

[12] US Environmental Protection Agency (2003). *Environmental and Health Assessment of Perfluoro Sulfonic Acid and Its Salts*.

[13] Ding G., Peijnenburg W. J. G. M. (2013). Physicochemical properties and aquatic toxicity of poly- and perfluorinated compounds. *Critical Reviews in Environmental Science and Technology*.

[14] Jahnke A., Berger U. (2009). Trace analysis of per- and polyfluorinated alkyl substances in various matrices—how do current methods perform?. *Journal of Chromatography A*.

[15] Petrovic M., Farré M., de Alda M. L. (2010). Recent trends in the liquid chromatography-mass spectrometry analysis of organic contaminants in environmental samples. *Journal of Chromatography A*.

[16] Núñez O., Gallart-Ayala H., Martins C. P. B., Lucci P. (2012). New trends in fast liquid chromatography for food and environmental analysis. *Journal of Chromatography A*.

[17] Takino M., Daishima S., Nakahara T. (2003). Determination of perfluorooctane sulfonate in river water by liquid chromatography/atmospheric pressure photoionization mass spectrometry by automated on-line extraction using turbulent flow chromatography. *Rapid Communications in Mass Spectrometry*.

[18] Wilson S. R., Malerød H., Holm A., Molander P., Lundanes E., Greibrokk T. (2007). On-line SPE-nano-LC-nanospray-MS for rapid and sensitive determination of perfluorooctanoic acid and perfluorooctane sulfonate in river water. *Journal of Chromatographic Science*.

[19] Gosetti F., Chiuminatto U., Zampieri D. (2010). Determination of perfluorochemicals in biological, environmental and food samples by an automated on-line solid phase extraction ultra high performance liquid chromatography tandem mass spectrometry method. *Journal of Chromatography A*.

[20] Enevoldsen R., Juhler R. K. (2010). Perfluorinated compounds (PFCs) in groundwater and aqueous soil extracts: using inline SPE-LC-MS/MS for screening and sorption characterisation of perfluorooctane sulphonate and related compounds. *Analytical and Bioanalytical Chemistry*.

[21] Llorca M., Farré M., Picó Y., Müller J., Knepper T. P., Barceló D. (2012). Analysis of perfluoroalkyl substances in waters from Germany and Spain. *Science of the Total Environment*.

[22] Gode D., Martin M. M., Steiner F., Huber C. G., Volmer D. A. (2012). Rapid narrow band elution for on-line SPE using a novel solvent plug injection technique. *Analytical and Bioanalytical Chemistry*.

[23] Rodier D. R., Birks J. W. (1994). Dual injector solvent elution and focussing technique for the on-line analysis of solid-phase extraction cartridges in HPLC. *Chromatographia*.

[24] Tittlemier S. A., Braekevelt E. (2011). Analysis of polyfluorinated compounds in foods. *Analytical and Bioanalytical Chemistry*.

[25] Martin J. W., Kannan K., Berger U. (2004). Analytical challenges hamper perfluoroalkyl research. *Environmental Science and Technology*.

[26] Valsecchi S., Rusconi M., Polesello S. (2013). Determination of perfluorinated compounds in aquatic organisms: a review. *Analytical and Bioanalytical Chemistry*.

[27] Sojo L. E., Lum G., Chee P. (2003). Internal standard signal suppression by co-eluting analyte in isotope dilution LC-ESI-MS. *Analyst*.

[28] Liang H. R., Foltz R. L., Meng M., Bennett P. (2003). Ionization enhancement in atmospheric pressure chemical ionization and suppression in electrospray ionization between target drugs and stable-isotope-labeled internal standards in quantitative liquid chromatography/tandem mass spectrometry. *Rapid Communications in Mass Spectrometry*.

[29] Enke C. G. (1997). A predictive model for matrix and analyte effects in electrospray ionization of singly-charged ionic analytes. *Analytical Chemistry*.

[30] Cech N. B., Enke C. G. (2001). Effect of affinity for droplet surfaces on the fraction of analyte molecules charged during electrospray droplet fission. *Analytical Chemistry*.

[31] Valsecchi S., Rusconi M., Mazzoni M. (2014). Occurrence and sources of perfluoroalkyl acids in Italian river basins. *Chemosphere*.

